# Microchannel network hydrogel induced ischemic blood perfusion connection

**DOI:** 10.1038/s41467-020-14480-0

**Published:** 2020-01-30

**Authors:** Jung Bok Lee, Dae-Hyun Kim, Jeong-Kee Yoon, Dan Bi Park, Hye-Seon Kim, Young Min Shin, Wooyeol Baek, Mi-Lan Kang, Hyun Jung Kim, Hak-Joon Sung

**Affiliations:** 1 Department of Medical Engineering, Yonsei University College of Medicine, 50-1 Yonsei-ro, Seodaemun-gu, Seoul, 03722 Republic of Korea; 2 Department of Plastic & Reconstructive Surgery, Yonsei University College of Medicine, 50-1 Yonsei-ro, Seodaemun-gu, Seoul, 03722 Republic of Korea; 3 TMD LAB Co. Ltd., 50-1 Yonsei-ro, Seodaemun-gu, Seoul, 03722 Republic of Korea; 4 Department of Biomedical Engineering, The University of Texas at Austin, Austin, TX 78712 USA

**Keywords:** Implants, Peripheral vascular disease, Therapeutics

## Abstract

Angiogenesis induction into damaged sites has long been an unresolved issue. Local treatment with pro-angiogenic molecules has been the most common approach. However, this approach has critical side effects including inflammatory coupling, tumorous vascular activation, and off-target circulation. Here, the concept that a structure can guide desirable biological function is applied to physically engineer three-dimensional channel networks in implant sites, without any therapeutic treatment. Microchannel networks are generated in a gelatin hydrogel to overcome the diffusion limit of nutrients and oxygen three-dimensionally. Hydrogel implantation in mouse and porcine models of hindlimb ischemia rescues severely damaged tissues by the ingrowth of neighboring host vessels with microchannel perfusion. This effect is guided by microchannel size-specific regenerative macrophage polarization with the consequent functional recovery of endothelial cells. Multiple-site implantation reveals hypoxia and neighboring vessels as major causative factors of the beneficial function. This technique may contribute to the development of therapeutics for hypoxia/inflammatory-related diseases.

## Abstract (web summary)

Restoration of blood flow to damaged sites has commonly involved treatment with pro-angiogenic molecules but these have undesired side effects. Here the authors present a microchannel-patterned gelatin hydrogel that is able to rescue mouse and porcine models of hindlimb ischemia.

## Introduction

Blood vessel growth into damaged sites or implanted scaffolds is a pre-requisite process for tissue regeneration^1,2^. Local treatment with pro-angiogenic molecules such as vascular endothelial growth factor (VEGF) has been considered the most promising approach^3–5^. The molecule is injected to target sites, released from implants, or delivered via systemic circulation^6–10^. However, critical limitations to this approach include unexpected side effects influenced by off-target sites and biomolecular treatment. Blood or interstitial flow, as the primary route for molecules to reach target sites, often guides their delivery into systemic circulation^11^. VEGF induces angiogenesis as well as inflammation, and over-induction of angiogenesis may lead to tumorous progression^12^. Although a promising strategy was reported to address this issue by enabling sustained and low-dose delivery of VEGF with fibrin biomaterials^13^, the chronic issues in pro-angiogenic molecular treatment remain unsolved, and urgently require an advance in the concept of most current approaches.

As an unexplored solution, vascular networks can be physically engineered in damaged sites following the concept that structure guides biological function. Surface patterning, porous scaffold design, and 3D bioprinting have been widely studied in the past decade to control cell functions^14–18^. To become functional, vasculatures require the following unique structures: (i) a three-dimensionally closed network to enable loss-free perfusion from inlet to outlet points, (ii) branched connections to cover the maximum surface area with minimum flow resistance, (iii) high vessel packing to overcome the 200 µm diffusion limit of oxygen and nutrients in an omnidirectional manner, and (iv) extracellular matrix scaffolding to facilitate molecular diffusion to intravascular spaces and to support in-and-out microvessel growth. Blood flows preferably through a vascular network that is equipped with the aforementioned structures. These facts indicate that setting a functional vascular structure inside an implant may induce blood perfusion from the host tissue to the implant. Indeed, a previous study reported that angiogenesis occurred most in ~40 µm pore diameters with an interconnected inner structure in implantable scaffolds^14,15^.

The pathophysiological environment also affects angiogenic processes^19,20^. The neighboring blood vessels can grow into damaged sites (angiogenesis) whereas stem cells or monocytes can differentiate to endothelial progenitor cells (EPCs) even in the absence of blood vessels (vasculogenesis)^21,22^. These processes can be induced by local pathogenic events such as hypoxia and inflammation^23–25^. Under hypoxic conditions, the body operates homeostatic functions to overcome this status, resulting in the induction of angiogenesis. Monocytes can undergo activation or phenotype changes to macrophages, osteoclasts, or EPCs in response to specific pathophysiological signals^26^. Among these physiological monocyte functions, EPC differentiation and macrophage polarization to pro-inflammatory M1 or pro-angiogenic M2 play regulatory roles in angiogenesis versus vasculogenesis^27–29^. Because both EPC and M2 polarization induce the formation of a vascular network^26,27,30^, pre-setting the functional vascular structure may conversely promote these pro-angiogenic monocyte functions.

This study examines the induction of blood perfusion to damaged sites by implanting a hydrogel containing microchannel networks to obtain functional vascular structures. The promising therapeutic effect of hydrogel is demonstrated in mouse and porcine models of hindlimb ischemia. Its functional vascular structure with microchannel size results in promoted pro-angiogenic M2 polarization of macrophages and consequent functional endothelial cell (EC) recovery.

## Results

### Channel network hydrogel fabrication

**Fig. 1:**
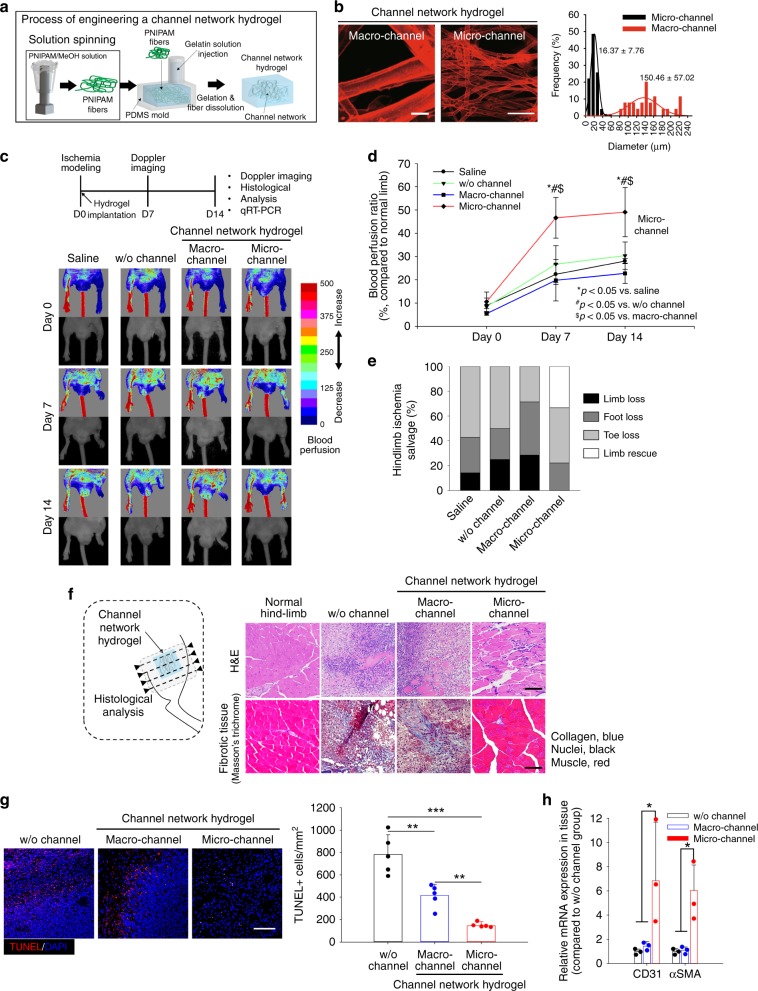
Implantation of microchannel network hydrogel in mouse ischemic hindlimb tissue. **a** Schematic illustration of the procedure to produce poly(*N*-isopropylacrylamide) (PNIPAM) fibers, then channel networks, in a hydrogel within a PDMS mold. **b** Confocal visualization of micro- or macrochannel networks in hydrogels with their channel diameter distribution. Channels were perfused with FluoSpheres (45 nm, red). Scale bar = 100 μm. **c** Laser Doppler perfusion imaging (LDPI) of supine position in a mouse model of hindlimb ischemia with **d** quantification of the corresponding blood perfusion ratio, compared to that of normal hindlimb at days 0, 7, and 14 post-implantation (*N* = 5). Statistical significances are determined using one-way ANOVA with Tukey post-hoc pairwise comparisons; *p < 0.05 versus saline; ^#^*p* < 0.05 versus without (w/o) channel; and ^$^*p* < 0.05 versus macrochannel group. **e** Fraction ratio of limb salvage in ischemic hindlimb at day 14 post surgery. **f** General histology (H&E: top) and fibrotic tissue staining (Masson’s trichrome: bottom) of distal hindlimb tissue site from the implant. Scale bar = 100 μm. **g** Cell apoptosis in hindlimb tissue at the distal site from the implant by terminal deoxynucleotidyl transferase (TdT) dUTP nick-end labeling (TUNEL) assay with quantitative analysis (*N* = 5). Scale bars = 100 μm; **p < 0.01 and ***p < 0.005 between lined groups. **h** Gene expression of CD31 and alpha smooth muscle actin (αSMA) at the distal tissue site from the implant by qRT-PCR (*N* = 3). **f**–**h** Dots represent each animal. Data presented are mean ± SEM. Statistical significances are determined using one-way ANOVA with Tukey post-hoc pairwise comparisons; *p < 0.05 between lined groups. Source data are provided as a Source Data file.

3D channel network hydrogels were produced through the following process: (i) poly(*N*-isopropylacrylamide) (PNIPAM) was solution-spun to produce fibers (Fig. 1a and Supplementary Fig. 1). PNIPAM was chosen owing to its biocompatibility and thermo-responsibility (lower critical solution temperature (LCST) = 32 °C). (ii) PNIPAM fibers were embedded in a polydimethylsiloxane (PDMS) mold, then an enzyme-crosslinkable gelatin solution was poured onto them, followed by gelation cross-linking. (iii) The temperature-dependent water-solubility of PNIPAM enabled the fibers to dissolve by washing with water perfusion when the temperature was lowered below 32 °C, representing a non-toxic, organic solvent-free process. In this way, void channel networks were generated post fiber dissolving and perfusion washing (Supplementary Figs. 1 and 2). In our previous study, the washing process enabled complete removal of PNIPAM fibers throughout the channel gel when analyzed by digital imaging and high-performance liquid chromatography (HPLC)^31^.

To determine the effect of channel size, micro- and macro-diameters were produced by controlling PNIPAM fiber diameter. The resultant channel structure and interconnectivity were analyzed post FluoSpheres (45 nm, red) perfusion, followed by confocal imaging because only the interconnected channels allowed for perfusion staining (Fig. 1b and Supplementary Fig. 3). The average diameters of micro- and macrochannels were 16.37 ± 7.76 and 150.46 ± 57.02 µm (mean ± SEM), respectively. The microvasculature-like diffusion and perfusion characteristics of the channel network were demonstrated (Supplementary Fig. 4). As a result, fluorescein isothiocyanate (FITC)-labeled dextran (40 kDa, green) diffused efficiently from the microchannels into the gelatin matrix in the hydrogel, whereas microbeads (45 nm FluoSpheres, red) perfused well through the microchannels. As the lead-off trial to vascularize damaged tissue using only the channel network structure of hydrogel, the standard mouse model of hindlimb ischemia^32^ was modified to result in several ischemic damages and consequent limb amputation (Supplementary Fig. 5a). The two (up and down) points of femoral vessels were ligated to minimize the spontaneous rescue effect of collateral formation. Then, right after surgery, a test hydrogel was implanted in the center of the severe ischemic region between the two ligation points. In the rescue process developed in this study, the proximal and distal host vessels grew into the hydrogel, and were followed by perfusion connection with the microchannel network (Supplementary Fig. 5b). This process enabled blood perfusion from the proximal host vessels (inlet) to the microchannels, and then to the distal vessels (outlet), as in the closed circulation system of the body, thereby rescuing the ischemic limb tissue damage. The following results prove the developed rescue mechanism.

In the mouse model of severe hindlimb ischemia (Supplementary Fig. 5a), blood perfusion degrees (Fig. 1c, d) in the hindlimb with the resulting limb salvage (Fig. 1e) were examined by laser Doppler perfusion imaging (LDPI) with quantitative analysis at days 0, 7, and 14 post-implantation of the test hydrogels. Severe ischemia modeling was successfully induced in the ligated left hindlimb of each group with a 5–10% blood perfusion ratio to that of the normal right limb at day 0 (Fig. 1c, d). Overall, implantation of the microchannel gel enhanced the perfusion ratio by up to 50% at days 7 and 14, while the normal right limb maintained a 100% perfusion ratio until day 14. In contrast, the control (saline and without (w/o) channel) and macrochannel groups showed progressive limb loss due to the lack of sufficient perfusion (<30% perfusion ratio) from days 7 to 14. As a result (Fig. 1e), there was no limb loss with significant limb rescue in the microchannel group, whereas the other groups showed losses of the toe, foot, or limb with the lack of limb rescue.

Next, histological examination with hematoxylin and eosin (H&E) and Masson’s trichrome staining, along with TUNEL assay, were conducted using tissue samples from the distal site of the implant at day 14 post-implantation (Fig. 1f, g). The macro- and w/o channel groups showed invasion of massive inflammatory cells with formation of severe fibrotic tissues, whereas the microchannel group showed significant restoration of muscle tissue to the level of a normal limb, with a normal-like population of inflammatory cells (Fig. 1f). These results were supported by the pattern of cell death, in which a significantly smaller number of TUNEL^+^ cells were present in the microchannel group compared to those in the other groups (Fig. 1g). Moreover, the gene expression of CD31 and αSMA in the microchannel group was higher than those of the other test groups, indicating a causative role of the microchannel in promoting functional vessel ingrowth (Fig. 1h).

### Vessel ingrowth with perfusion connection

**Fig. 2:**
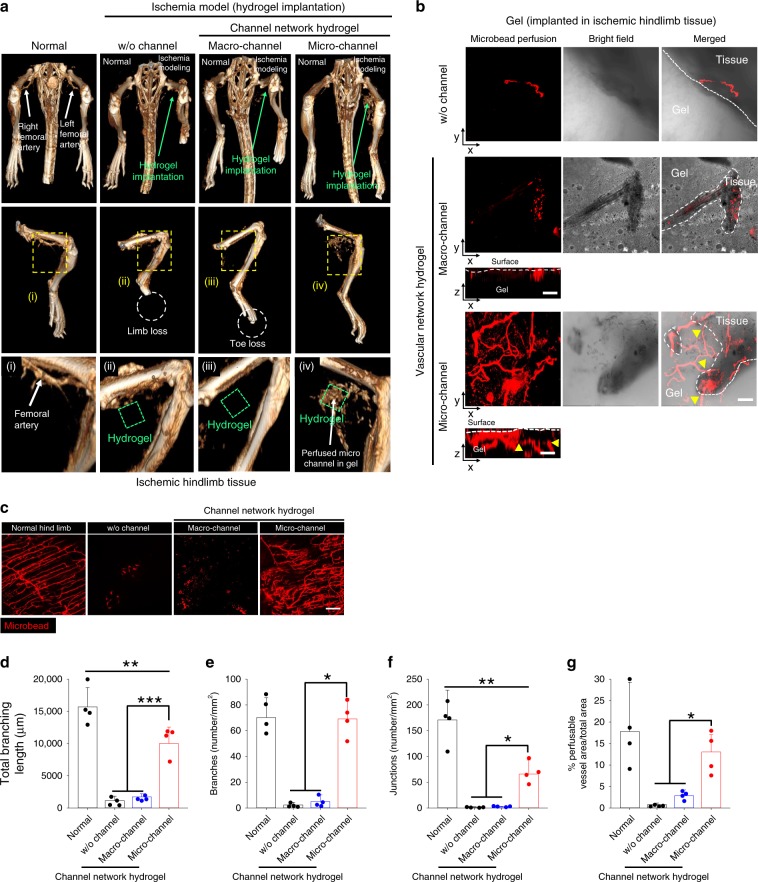
Promotion of host vessel ingrowth and perfusion connection with channel networks. **a** MicroCT images of arterial vasculature in ischemic tissue of mouse hindlimb at day 14 post hydrogel implantation (green dot box). **i**–**iv** Middle and bottom rows: high magnification images of the hydrogel implantation sites. Confocal images of harvested **b** whole channel network hydrogel and **c** hindlimb tissues post-perfusion of red FluoroSpheres, through the left ventricle at day 14 indicate ingrowth (yellow arrows) of host blood vessels and perfusion connection with the microchannel network (red). Scale bars = 100 μm. Quantification of microvasculature structural parameters: **d** total branching length, **e** branch number, **f** junction number, and **g** perfusable vessel area per field of view (FOV) at ischemic hindlimb tissue (*N* = 4). Dots represent each animal. Data presented are mean ± SEM. Statistical significances are determined using one-way ANOVA with Tukey post-hoc pairwise comparisons; *p < 0.05, **p < 0.01, and ***p < 0.005 between lined groups. Source data are provided as a Source Data file.

The primary mechanism through which the severely damaged limb is rescued by the microchannel hydrogel was investigated. First, the perfusion connection between the host vessels and the microchannel network was examined by micro-computed tomography (microCT) and perfusion-based confocal imaging with quantitative analysis at day 14 post-implantation (Fig. 2a–d) in the mouse model of severe hindlimb ischemia. In the microCT images with gradual increases in the magnification (from the top to bottom rows), the microchannel group showed clear microvessel invasion from the host femoral artery into the hydrogel in contrast to the other test groups (Fig. 2a). As a zoom-in support, FluoSphere (red) perfusion-based confocal images showed clear host vessel ingrowth and perfusion connection with the microchannel network of the implanted hydrogel, whereas the red perfusion signal was absent in the other test groups, indicating the lack of the vessel ingrowth (Fig. 2b). Structural characterization of the formed vasculature (Fig. 2c) with quantitative analysis (Fig. 2d–g) also supported these results. As indicators of the functional vascular structure, the total branching length, number of branches and junctions, and fractional area in the microchannel hydrogel group were significantly higher than those in the other test groups, reaching a level similar to that of the normal limb. The branching parameters were measured, as they were essential indicators of blood vessel functionality. As blood vessel branching increases, the covered surface area increased, thereby enhancing the delivery efficiency of oxygen and nutrient to every corner of the tissues^33,34^. This branched structure also reduces flow resistance by mimicking the parallel connection of electric circuits^35,36^.

### Macrophage infiltration and polarization

**Fig. 3:**
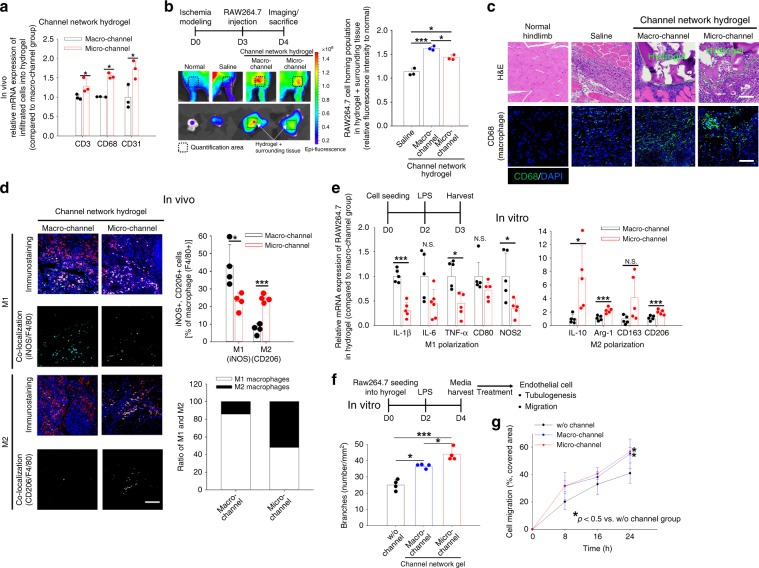
Monocyte/macrophage responses to test groups in a mouse model of hindlimb ischemia. **a** Gene expression (CD3, CD68, and CD31) of infiltrated cells into macro- versus microchannel network hydrogels only after removing surrounding tissues at day 14 post-implantation by qRT-PCR (*N* = 3). Data presented are mean ± SEM. Statistical significances are determined using a two-tailed Student’s *t*-test; *p < 0.05 between lined groups. **b** IVIS images of monocyte infiltration into the implant site (hydrogel + surrounding tissue) of each test group on day 1 post intravenous administration of Vivotrack680-labeled RAW264.7 cells, followed by quantification of the ratio in ischemic to normal hindlimb (*N* = 3). Data presented are mean ± SEM. Statistical significances are determined using one-way ANOVA with Tukey post-hoc pairwise comparisons; *p < 0.05 and ***p < 0.005 between lined groups. **c** Representative H&E and immunostaining (CD68) images of ischemic hindlimb tissues at the implant site. Scale bar = 100 µm. **d** Confocal images of macrophage polarization markers (M1: iNOS versus M2: CD206, both in green), a mouse macrophage marker (F4/80 in red), and nucleus (DAPI in blue) at day 14 post-implantation. The corresponding M1 or M2 cell number % out of the total macrophage number (F4/80^+^) as well as M1/M2 ratios in the macro- and microchannel groups were determined quantitatively. Scale bar = 100 µm. Data presented are mean ± SEM. Statistical significances are determined using a two-tailed Student’s *t*-test; *p < 0.05 and ***p < 0.005 between lined groups (*N* = 4). **e** Gene expressions of M1 (IL-1β, IL-6, TNF-α, CD80, and NOS2) and M2 (IL-10, arginase-1, CD-163, and CD-206)] markers in mouse macrophages (RAW264.7) post-culture within macro- versus microchannel network hydrogels in vitro by qRT-PCR (*N* = 5). Data presented are mean ± SEM. Statistical significances are determined using a two-tailed Student’s *t*-test; *p < 0.05 and ***p < 0.005 between lined groups (N.S.: not significant). Data presented are mean ± SEM. Quantification of endothelial cell (human umbilical vein endothelial cell, HUVEC) **f** tubulogenesis and **g** migration induced conditioned media of RAW264.7 cells in vitro (*N* = 4). Dots represent each replicate of HUVEC-seeded wells in a 24-well plate. Data presented are mean ± SEM. Statistical significances are determined using one-way ANOVA with Tukey post-hoc pairwise comparisons; *p < 0.05 and ***p < 0.005 between lined groups. Source data are provided as a Source Data file.

The binary roles of monocytes in regulating host responses have been widely studied^29,37^. In particular, their polarization to destructive M1 and regenerative M2 phenotypes in response to hydrogel channel size was examined at day 14 post-implantation. Inflammatory cells infiltrated the microchannel hydrogel implants significantly more than the other test groups. This was indicated by the gene expression of representative T cell (CD3) and monocyte/macrophage markers (CD68), in addition to CD31 in the implanted hydrogels after removing the surrounding tissues (Fig. 3a). Because it is not possible to track inflammatory cell homing into the implanted sites in real time, RAW264.7 mouse monocytes were labeled with Vivotrack-680 and injected into mice via the tail vein at day 3 post-implantation surgery. This was followed by imaging via in vivo imaging system (IVIS) at 24 h post cell injection (Fig. 3b). The population of RAW264.7 mouse monocytes homing into the implanted hydrogels and surrounding tissues was significantly larger in the macrochannel group compared to the microchannel and saline groups. As shown by H&E histology and CD68 immunostaining of tissues harvested around the implants, more macrophages were observed in the channel hydrogel groups than in the normal and saline groups (Fig. 3c). The CD68^+^ populations (Fig. 3a) in the hydrogels only (as opposed to the homing populations of RAW264.7 mouse monocytes (Fig. 3b) in the hydrogels and surrounding tissues) indicated a positive effect of microchannel size on CD68^+^ cell infiltration into the implanted hydrogels without surrounding tissues.

In this situation, destructive M1 and pro-angiogenic M2 polarization were determined by immunostaining of iNOS (M1) and CD206 (M2) in the implant sites at day 14 post-implantation (Fig. 3d and Supplementary Fig. 6). M2 polarization of macrophages was more dominant in the microchannel group than in the macrochannel group, contrary to the M1 polarization pattern, indicating an M2 polarization mediator in the microchannel hydrogel rescue mechanism. We also examined the channel size-dependent monocyte polarization in vitro. RAW264.7 cells were M1-stimulated with lipopolysaccharide (LPS) in macro- and microchannel network hydrogels. When the activated macrophages were cultured inside the channel groups for 4 days under media perfusion, the adhered, spindle cell shapes were dominant in the macrochannels in contrast to the circular, monocyte-like morphologies in the microchannels, indicating attenuation of M1 macrophage polarization by microchannels (Supplementary Fig. 7). This result was supported by the quantitative real-time polymerase chain reaction (qRT-PCR) data (Fig. 3e), in which the gene expression of M2 markers (IL10, Arg-1, CD163, and CD206) was significantly higher in the microchannel group than in the macrochannel group as opposed to the expression pattern of M1 markers (IL-1β, IL-6, TNF-α, CD80, and NOS2). Compared to the w/o channel group, the two channel groups increased the in vitro M2 polarization of macrophages, as evidenced by increased EC migration and tubulogenesis under treatment of the conditioned media from culture of RAW264.7 cells with LPS stimulation (Fig. 3f, g). In particular, the degree of tubulogenic branching in the microchannel group was significantly higher than that of the rest groups, confirming the pro-angiogenic effect of microchannel size (Fig. 3f).

### Endothelial cell infiltration and ingrowth

**Fig. 4:**
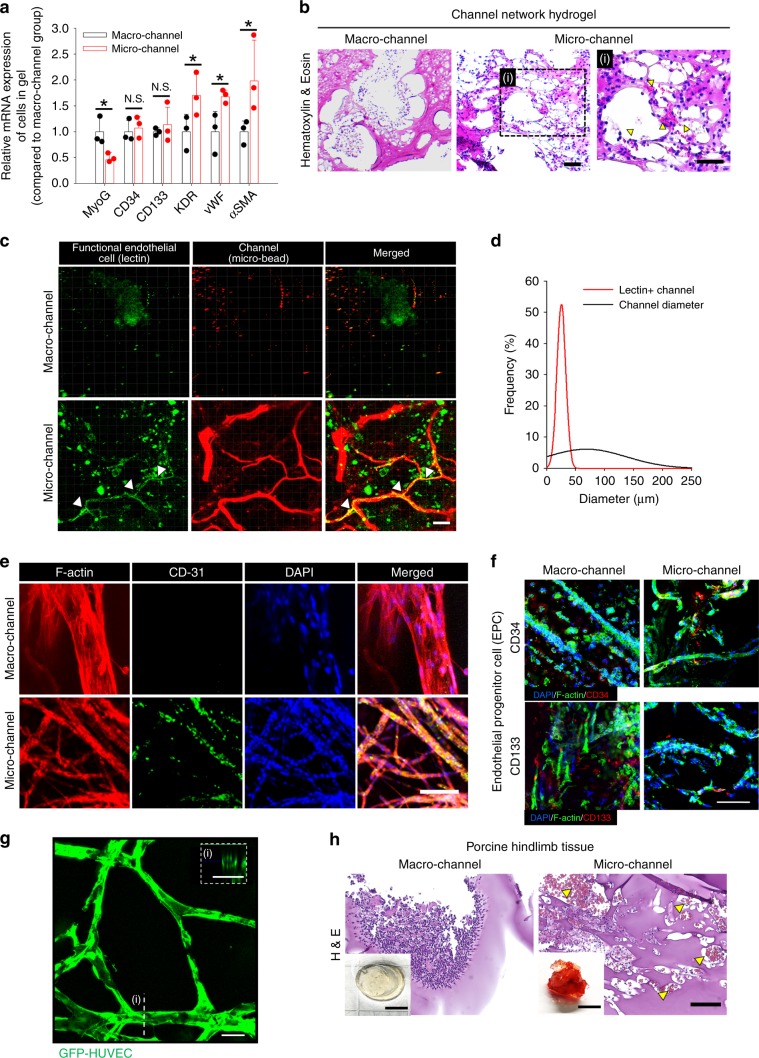
In vivo infiltration and ingrowth of ECs within channel network hydrogels. EC infiltration and ingrowth within channel network hydrogels from 2-week implantation in mouse (**a**–**f**) and porcine (**h**) models of hindlimb ischemia. **a** Gene expression of infiltrated cells between macro- and microchannel groups at day 14 post-implantation (*N* = 3). Dots represent each replicate. Data presented in mean ± SEM. Statistical significances are determined using two-tailed Student’s *t*-test; *p < 0.05 between lined groups (N.S.: not significant). **b** H&E images of implanted channel network hydrogels. In the box with high magnification (right, i), yellow arrows indicate sites where blood cells infiltrated the microchannel network hydrogel. Scale bar = 100 µm. **c** Confocal images of functional ECs (lectin^+^), in macro- and microchannel groups and **d** quantitative analysis of lectin^+^ channel size distribution. The images were obtained from whole-mount hydrogels post-EC staining (green, lectin) and channel perfusion (red, microbeads). Scale bar = 100 µm. White arrows indicate co-localization of functional ECs (green) and perfusion-stained channel (red). **e** Confocal images of whole-mount hydrogel with macro- or microchannel network post co-immunostaining of CD31 (green), F-actin (red), and nucleus (blue). Scale bar = 100 µm. **f** Confocal images of whole-mount hydrogel with macro- or microchannel network post co-immunostaining of CD34/CD133 (red) and F-actin (green) with nucleus (blue). Scale bar = 100 µm. **g** Confocal image of ECs (GFP-HUVEC) lining a microchannel network in vitro (i: a cross-sectionaview of EC-lined channel). Scale bar = 100 µm. **h** H&E images of implanted channel network hydrogels in porcine hindlimb ischemia. Yellow arrows indicate the sites where blood cells infiltrated in the microchannel group. Scale bars = 5 mm for digital images and 100 µm for H&E images. Source data are provided as a Source Data file.

As pro-angiogenic M2 polarization of macrophages with CD31^+^ cell infiltration was dominant in the microchannel network hydrogel (Fig. 3), the consequent behavior of ECs was examined (Fig. 4). The gene expression for EC markers (KDR and vWF) and a vascular smooth muscle cell marker (αSMA) was significantly higher in the microchannel group than in the macrochannel group (Fig. 4a). EPC infiltration into the micro- and macrochannel groups was confirmed by the gene (Fig. 4a) and protein (Fig. 4f) expression of the EPC marker (CD34 and CD133). The gene expression of a skeletal muscle marker (MyoG) was higher in the macrochannel group than in the microchannel group, indicating an invasion of skeletal muscle with increased channel size.

These results were supported by the increases in blood cell appearance (Fig. 4b, h) and blood spreading (digital images in Fig. 4h and Supplementary Fig. 6) of the microchannel group compared those of the macrochannel group in the mouse and porcine models of hindlimb ischemia. Indeed, markedly more functional ECs (Lectin^+^) infiltrated the microchannel than the macrochannel, as analyzed by immunostaining (Fig. 4c) with quantitative profiling of Lectin^+^ cell population and channel diameter (Fig. 4d). Microchannel-guided vessel ingrowth was confirmed by marker expression of ECs (CD31) and EPCs (CD34 and CD133) by immunostaining (Fig. 4e–f). In addition, when GFP-HUVECs were cultured within the channel network hydrogels under media perfusion, in vitro microchannel endothelialization was more efficient than in the macrochannels, as presented by EC lining, tight junction, and capillary-like sprouting (Fig. 4g and Supplementary Fig. 8 and Movie 1). Together, the overall interpretation of the results (Figs. 3 and 4) suggests a causative role of M2 macrophage polarization in promoting EC infiltration and ingrowth as a mechanism guiding host vessel ingrowth and perfusion connection with microchannel networks.

### Wound healing model

**Fig. 5:**
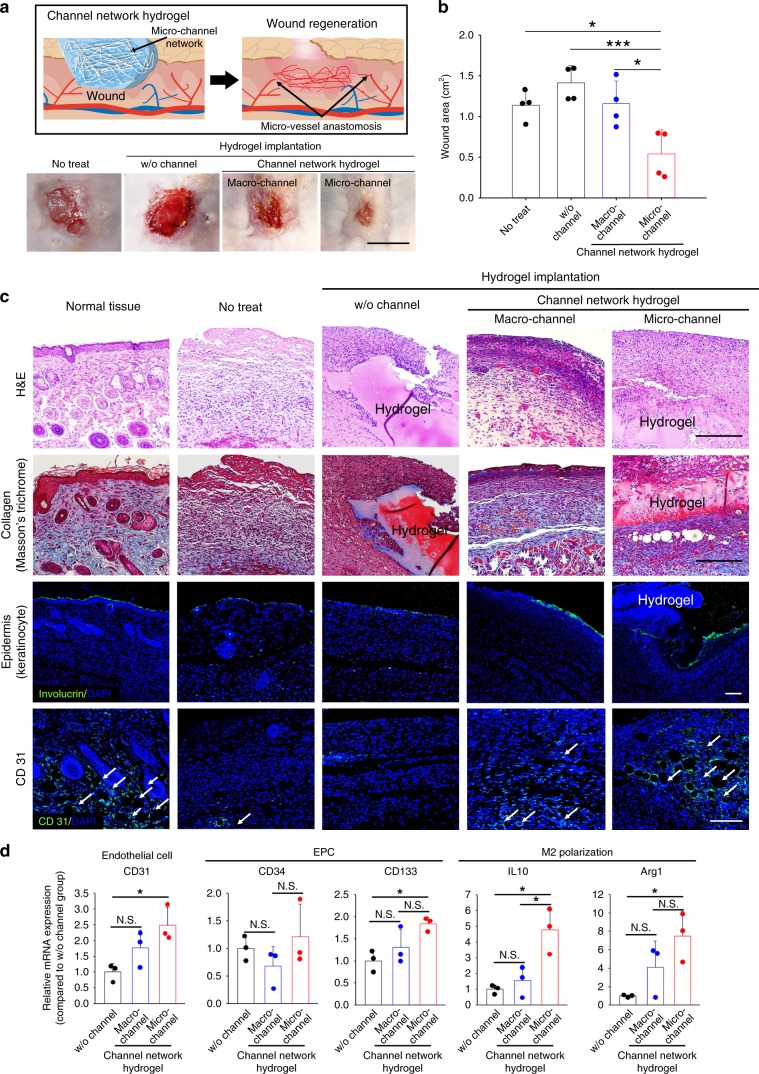
Regenerative effects of test hydrogels on wound closure. **a** Schematic illustration of hydrogel implantation into a wound site post-full-thickness defect of mouse dorsal skin with the discovered regeneration process (top box). Photographs of wound healing sites at day 14 post-implantation (bottom row). Scale bar = 1 cm. **b** Degrees of decreased wound area from the initial 2 × 2 cm defect in each group at day 14 post-implantation (*N* = 4). Dots represent each animal. Data are presented as mean ± SEM. Statistical significances are determined using one-way ANOVA with Tukey post-hoc pairwise comparisons; *p < 0.05 and ***p < 0.005 between lined groups. **c** Representative images of general histology (H&E), collagen formation (Masson’s trichrome), keratinocyte-epidermis lining (green Involucrin), and CD31^+^ cells (green) with nuclei (blue DAPI). White arrows point out microvascular structures (CD31+) in the skin tissue sections. Scale bar = 100 µm. **d** Marker gene expression of EC (CD31), EPC (CD34 and CD133), and M2 polarization (IL10 and Arg1) in the wound area of mouse dorsal skin at day 14 post-implantation by qRT-PCR (*N* = 3). Dots represent each animal. Data are presented as mean ± SEM. Statistical significances are determined using one-way ANOVA with Tukey post-hoc pairwise comparisons; *p < 0.05 between lined groups (N.S.: not significant). Source data are provided as a Source Data file.

To examine whether the microchannel network hydrogel can be applied for another model of ischemic damage where neighboring host vasculature is present, the test groups were implanted in a well-established mouse model of wound healing (Fig. 5). As demonstrated in the hindlimb ischemia model, neighboring vessels might grow into the hydrogel and undergo a perfusion connection with the microchannel network. Indeed, implantation of the microchannel group accelerated the wound closure process in the wound area compared to the macrochannel group and the w/o channel hydrogel group. This is visualized (Fig. 5a and Supplementary Fig. 9) and quantitatively analyzed (Fig. 5b) in our figures. This result was supported by more robust tissue formation (H&E), richer collagen content (Masson’s trichrome staining), and accelerated epidermis formation (keratinocyte staining) in the microchannel and normal tissue groups compared to the rest groups (no-treat, w/o channel, and macrochannel) (Fig. 5c). Moreover, the microchannel group promoted infiltration of ECs and EPCs in the wound area compared to the w/o channel group as shown by their marker staining (CD31 and CD133, respectively). Interestingly, the expression level of another EPC marker, CD34 was not significantly different between the macro- and microchannel groups (Fig. 5d), indicating potential, non-specific infiltration of other CD34^+^ cell types such as bone marrow cells by both channel groups. M2 polarization also increased in the microchannel group compared to the w/o channel group as presented by the amount of section factors (IL10 and Arg1). CD 31 expression (EC marker) and IL10 secretion (M2 polarization) were higher in the microchannel group compared to the macrochannel group but the differences of other factor amounts between the macro- and microchannel groups were not significant, indicating relative difficulty to discern the channel size effect in the wound healing model compared to the severe hindlimb ischemia model.

### Non-ischemic in vivo models

**Fig. 6:**
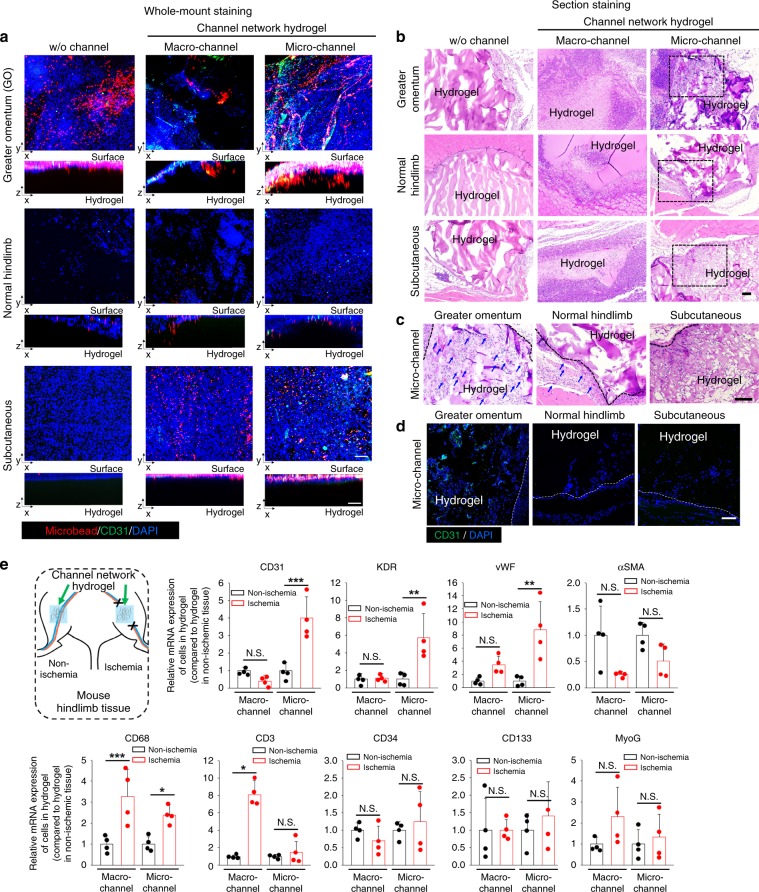
Vascularization efficiencies of microchannel hydrogel implantation in non-ischemic mouse models. The number of surrounding host vessels decreases from the greater omentum to normal hindlimb, and lastly, to the subcutaneous site. **a** Confocal images of whole-mount hydrogels post perfusion of blood vessels (red fluorescence microbeads) with immunostaining of CD31^+^ cells (green) and nucleus (blue DAPI) at day 14 post-implantation. The images were three-dimensionally reconstructed to determine vascular ingrowth and perfusion connection through red FluoroSphere perfusion from host vessels into the channel network. Scale bar = 100 µm. **b** Representative H&E images of test groups. The box areas (100×) were magnified to **c** (200×). Scale bar = 100 µm. **c** High magnification (200×) H&E images from black-dotted boxes of micro-channel group in **b**. Blue arrows indicate the points where blood cells infiltrated into the implanted hydrogel or surrounding tissue of the greater omentum, normal hindlimb, and subcutaneous site (no blood cell infiltration). Scale bar = 100 µm. **d** Confocal images of CD31^+^ cells (green) in the sectioned tissues of each model containing a part of microchannel network hydrogel (blue: nucleus with DAPI staining). Scale bar = 100 µm. **e** Effects of channel size on cell infiltration into the normal side versus the ischemic hindlimb side, as presented by marker gene expression of the vessel (CD31, KDR, vWF, and αSMA), skeletal muscle (MyoG), monocyte/macrophage (CD68), T cell (CD3), and EPC (CD34 and CD133) cells in macro- and microchannel network hydrogels. qRT-PCR was conducted using only gel samples after removing the surrounding tissues on day 14 post-implantation (*N* = 4). Dots represent hydrogel sample in each animal. Data are presented mean ± SEM. Statistical significances are determined using two-tailed Student’s *t*-test; *p < 0.05, **p < 0.01, and ***p *<* 0.005 between lined groups (N.S.: not significant). Source data are provided as a Source Data file.

Given that both hindlimb ischemia and wound closure models are exposed to insufficient oxygen supply with a rich presence of neighboring host vessels, three other in vivo models (implantation to the omentum, normal limb, and subcutaneous tissue) were employed to determine the mechanistic roles of the ischemia condition and neighboring blood vessels. These three models are not subjected to ischemia, but the number of neighboring vessels decreased from rich (omentum), to middle (normal limb), and finally to poor (subcutaneous) levels. As demonstrated in perfusion-based (red FluoroSpheres) confocal images with CD31 staining (green) (Fig. 6a), (i) the microchannel group showed the most effective perfusion from host vessels to the implanted sample and the richest population of CD31^+^ cells; (ii) however, these levels in the microchannel group decreased markedly from the omentum model to the normal limb, and lastly, to the subcutaneous model; and (iii) even though the perfusion performance was most efficient in the microchannel group of the omentum model among all non-ischemic test groups, its level appeared to be lower than that of the microchannel group in the hindlimb ischemia model. These results were supported by the degrees of tissue ingrowth and blood cell infiltration in H&E images (Fig. 6b) with a comparison focused only on the microchannel groups (Fig. 6c) and CD31^+^ cell infiltration (Fig. 6d). In the three models, the w/o channel and macrochannel groups showed only a few or no CD31^+^ cells (Supplementary Fig. 10). The results indicate mechanistic roles of the ischemic condition and neighboring blood vessels in guiding the microchannel effect. Furthermore, the assistance to channel size effect was compared for the ischemic versus normal condition using the marker gene expression of infiltrated cells in the implanted hydrogels on day 14 post-implantation (Fig. 6e). The ischemic condition assisted the microchannel effect most effectively on vessel infiltration (CD31, KDR, vWF, and αSMA), whereas there was no noticeable effect of channel size and ischemic condition on infiltration of skeletal muscle (MyoG) and EPC (CD34 and CD133). Interestingly, regardless of the channel size, the ischemic condition promoted increased infiltration of monocyte/macrophage (CD68) and T cells (CD3) into the implanted hydrogels when compared to the normal side.

## Discussion

This study developed an implantable hydrogel with a perfusable microchannel network to treat ischemic/inflammatory disease by promoting angiogenesis and M2 polarization of the infiltrated monocytes/macrophages. We believe that this study significantly contributes to the field of regenerative medicine, in particular to the biotechnology areas where significant efforts have been made over the past decades to vascularize damaged tissues or implantable scaffolds. The study is based on the idea that a pre-engineered 3D microchannel network enabled vascular perfusion throughout the hydrogel implant via the ingrowth of neighboring host vessels and a perfusion connection with microchannels. Notably, this regenerative function was programmed by only the microchannel structure without loading any cells or therapeutic molecules. The microchannel size (16.37 ± 7.76 µm) and closed 3D network played a crucial role in guiding this regenerative mechanism in the gelatin hydrogel implantation^14,15^. The microchannel size was set up first to mimic the diameter range of capillary (5–10 µm), a small blood vessel^38^. Second, our previous studies proved that this diameter range of pores promoted micro blood vessel formation into the polymer’s scaffolds when implanted subcutaneously in mice^39^ while also accelerating the histological and functional recoveries of post hepatectomy liver via implantation of the same vascular network hydrogel that also contained minced pieces of autologous liver tissue in rabbits^31^. Lastly, a study reported that ~15 µm diameter pores induced M2 polarization and angiogenic activities^40^. The microchannel gel enabled perfusion from the upper stream to the implanted gel and beyond, even to the lower extremity (Fig. 1c), which served as an indirect indication of longitudinal blood flow. While this interpretation is macroscopic, the ingrowth and perfusion connection of host vessels were not only longitudinal but also of random direction as shown by microCT and fluorescent bead imaging (Fig. 2). Since the main outlet point of the microchannel is downstream of ligated femoral vessels, it is assumed that random directional microflow likely passed through the implanted hydrogel and collectively flew into the downstream outlet point through the perfusion connection, resulting in the longitudinal flow direction observed under the macroscopic doppler imaging (Fig. 1c).

The idea to generate a 3D-interconnected structure of sacrificial fibers originated from the cotton candy machine, because electrospinning did not work in depositing the volumetric fiber mesh, and 3D printing was not able to reach sufficient resolution for producing the closed network of dense fibers^41,42^. Only a cotton candy machine-like production of sacrificial fibers enabled nutrient diffusion limits to be overcome, which was a game-changer in this approach^43,44^. The use of enzyme cross-linkable gelatin hydrogel added practical value because it is cheap, biocompatible, and enables easy loading of cells and therapeutics before gelation. Moreover, its cross-linking time and degree are highly controllable by adjusting the enzyme to gelatin ratio, facilitating application to a wide range of tissues and organs^45,46^. Since microbial transglutaminase (mTG) cross-links glutamine and lysine via a transamidation reaction, the mechanical properties of the gelatin hydrogel depended on the mTG dosage and the amounts of these molecules as presented by the gelatin concentration. Previous studies reported that the gelation time of mTG gel ranged from ~15 s to hours with a constant mTG concentration (10 U g^−1^)^47–49^.

The microchannel hydrogel induced invasion and M2 polarization of monocytes (Fig. 3) and thereby promoted EC ingrowth from the host vessel side (Fig. 2) to localize into microchannels (Fig. 4), leading to the facilitation of blood perfusion (Fig. 1). Based on comparisons with the other test groups, the microchannel size was determined to play a key role in exerting this beneficial effect for the following reasons: (i) the saline control group showed a negligible effect on blood perfusion and limb rescue during 2-week implantation in the mouse hindlimb ischemia model (Fig. 1c). (ii) As an indication of the gelatin formulation effect, the gelatin hydrogel without channels (w/o channel group) demonstrated similar levels of negligible regenerative and pro-angiogenic effects (Figs. 1 and 2) to those of saline control group (Fig. 1c–e). (iii) When the same gelatin formation was kept, the reduction of channel size from the macrochannel group to the microchannel group resulted in remarkable promotion of beneficial effects throughout the study.

In this study, the microchannel function required hypoxic conditions and neighboring host vessels as a catalyst and a supporting reactor, respectively (Fig. 6). The evidence indicates that angiogenesis from host vessels made bridging of blood perfusion with microchannels and provided cellular and matrix sources for tissue regeneration. Promising results were demonstrated using a large porcine model, although the large size of the porcine vessels hampered the clear visualization of blood perfusion through the implantation site using current imaging tools. An inevitable issue in the use of pigs was their potent ability to induce spontaneous collateral formation and regenerate damaged tissues, even without any aid. This spontaneous vessel formation attenuated the response variation between ligated and unligated hindlimbs. Hence, a baboon or a dog model may be more suitable for future investigation. On the other hand, it can be assumed that the hydrogel porosity and degradability might be altered during the implantation period, as these are strongly interrelated, and may have thereby affected the results. When the porosity changes and degradability of hydrogels were determined in mice (post 2-week subcutaneous implantation) (Supplementary Fig. 11a, b), neither the porosity nor the degradability was significantly different between the macro- and microchannel groups. The degradability percentages of the channel groups were not significantly different from that of the w/o channel group (Supplementary Fig. 11b), indicating no significant effect of the porosity and degradability on the entire in vivo results. Also, regardless of channel size, the average pore sizes of macro- and microchannel groups increased to similar degrees after 2-week accelerated degradation in vitro. As a result, the difference of channel size between the macro- and microchannel group remained unaltered even after 2-week degradation, indicating non-significant effects of degradation and consequent pore size changes on the results (Supplementary Fig. 11c).

The transition from bench to clinical application requires an easy and fast fabrication process with scalable production. The production process of the microchannel network hydrogel needs only three simple steps (i.e., sacrificial fiber generation, hydrogel gelation with the fiber mesh embedding and removal). Moreover, as demonstrated in the large porcine model (Fig. 4h) and mouse wide-wound model (Fig. 5), the dimension of the channel hydrogel is scalable by changing the PDMS mold size and increasing the amounts of sacrificial fibers and gelatin hydrogel. With its inexpensive and easy production process, the regenerative effect of this microchannel network hydrogel is highly promising and applicable to peripheral artery disease (PAD) treatment. Considerable progress has been made in surgical and non-surgical treatments for patients with PAD, and there is still an unmet need to restore blood flow to ischemic tissues while avoiding detrimental inflammation and other side effects. Most strategies overlook the mechanistic role of the destructive inflammatory activation in PAD progression^50–52^. For this reason, the proposed approach can serve as an innovative platform for PAD treatment, as the microchannel network can induce pro-regenerative and pro-angiogenic monocyte polarization. Therefore, there is clear value in its transition toward clinical applications. The next version of the system will be designed to accelerate the perfusion connection to host vessels to enhance the clinical feasibility of the hydrogel. As one idea in a current trial, a central macrochannel line is formed in connection with surrounding microchannels so that the macrochannel can be surgically connected with host vessels post cannulation following the method discussed in a previous study^53^. In this case, the gel stiffness would be also adjusted to withstand the physiological pressure, and an anti-coagulant would be used to prevent thrombotic occlusions in the microchannel as demonstrated in our other recent study^54^. Although the current study is entirely focused on elaboration of the channel size as a key factor to enable ischemic blood perfusion connection, similar results may be obtained if the hydrogel is fabricated with same channel size using other materials. Therefore, this topic will be examined in the next step of study.

## Methods

### Device fabrication

As a sacrificial material to generate channel networks, PNIPAM (*M*_n_ = ~40,000, Sigma–Aldrich, St. Louis, MO), was dissolved in methanol at a concentration of 45% (weight volume^−1^). The fiber diameter was controlled by changing the spinning RPM in custom-built rotational spinning device^31,44^. The macro- and microfiber sizes were produced by spinning 45% PNIPAM/MeOH solution at 1000–1500 and 2500–2800 rpm, respectively. Next, PNIPAM fibers at a density of 11.45 ± 3.13 μg mm^−3^ were placed in the PDMS mold, and a silicone tube was placed to connect with the fibers at the inlet and outlet sides so that flow could be perfused through the inlet silicone tube to the fiber-generated channel network and to the outlet silicone tube. Then, a gelatin/mTG solution (9:1 ratio, final concentration = 5% weight volume^−1^) was poured onto it, followed by a cross-linking reaction with mTG at 37 °C. The embedded fibers were dissolved away from the mTG hydrogel by sol–gel transition of PNIPAM at room temperature with perfusing PBS in connection with the silicone tube (Supplementary Figs. 1a and 3). As a result, a 3D micro- or macrochannel network was formed in the gelatin hydrogel. The channel networks were visualized by perfusing red FluoroSpheres (45 nm, Invitrogen) and then imaged by confocal microscopy (LSM 780, Zeiss). The channel size distribution of the macro- and microchannels in the hydrogel was quantified using ImageJ/Fiji software. The channel network of test gelatin hydrogel was perfused with culture media containing FITC-dextran (molecular weight = 40,000, Sigma-Aldrich, MO) and red FluoroSpheres (45 nm, Invitrogen) throughout the channel network of test hydrogel at 20 μm min^−1^ for 30 min, followed by confocal imaging (LSM 780, Zeiss) to determine its diffusivity and perfusability, respectively.

### Mouse and porcine models of hindlimb ischemia

All procedures of mouse and porcine studies were approved by the Institutional Animal Care and Use Committee (IACUC) of Yonsei University College of Medicine (2016-0194 and 2017-0058 for mouse and porcine, respectively). For the mouse model, 5-week-old Balb/c male mice (Orient bio., Republic of Korea) with a weight range of 20–25 g were subject to anesthesia by intraperitoneal injection of xylazine (10 mg kg^−1^) and zoletil (50 mg kg^−1^). In their left limbs, the upper and lower points of femoral artery and vein were ligated using a 6-0 silk suture (Ethicon, Somerville, NJ)^32^ (Supplementary Fig. 5a), followed by resection of vessels between the two points. The ligation points were the proximal branch point of the external iliac artery and the distal point where it bifurcates into the saphenous and popliteal arteries. A test group hydrogel (4 mm × 4 mm × 3 mm) was implanted into the center hindlimb muscle of the ischemic vessel-resected area in a post-surgery mouse (Figs. 1–4) or the same muscle position of the non-ischemic hindlimb in a normal mouse (Fig. 6) to comparatively determine the ischemic effect until euthanized in 14 days. As another ischemia model in mice, a full thickness defect was induced on the dorsal skin of a mouse (Fig. 5). Then, a test group hydrogel (2 cm × 2 cm × 3 mm) was placed to cover the defect site by sealing with dressing film (Tegaderm^TM^, 3M Medical) for 14 days. As non-ischemic models, greater omentum and subcutaneous tissue with 14-day hydrogel implantation served as host neighboring vessel-rich and vessel-poor models, respectively, to determine the mechanistic role of the number of host vessels in angiogenesis-mediated perfusion connection with channel networks. Four mice were grouped to each wire-mesh cage ((W) 200 × (D) 260 × (H) 130 mm) and housed in a temperature (22 ± 2 °C) and humidity (50 ± 10%) regulated environment with a 12-h light–dark cycle.

For a porcine model of hindlimb ischemia, 3-month-old female Yorkshire pigs (XP Bio, Republic of Korea) of 40 kg were subject to intramuscular injection of atropine (0.04 mg kg^−1^), xylazine (2 mg kg^−1^), and azaperone (2 mg kg^−1^) as premedication. Anesthesia was then induced with Alfaxan (1 mg kg^−1^) and was maintained by endotracheal intubation of 2% isoflurane during surgery. As with the mouse model of hindlimb ischemia, the femoral artery and veins were ligated with 1-0 silk and dissected. Test group hydrogels (cylinder shape: 1 cm in diameter and 1.5 cm in height) were implanted into the central hindlimb muscle of the ischemic area until euthanized in 18 days for histological examination (H&E).

Because discrimination between the inside and outside of the hydrogel is only possible using histology, histological analysis was conducted on the hydrogel together with connected tissues immediately after harvesting. The hydrogel part was carefully dissociated from the connected tissue part, and the two parts were used separately to conduct quantitative PCR because positional discrimination was not possible using quantitative PCR (Supplementary Table 1).

### In vivo and in vitro degradability and porosity measurement

The in vivo degradability of the gelatin hydrogel groups (i.e., w/o channel, macrochannel, and microchannel in 1 cm diameter with 2 mm thickness) was determined by subcutaneously implanting into mice for 2 weeks (*N* = 4). Each test sample was then harvested, rinsed with PBS three times, and lyophilized to measure its dry weight. The mass loss as an indication of degradation degree was calculated by comparing to the corresponding dry weight before implantation (%). At the same time, the porosity of macro- or microchannel hydrogels was determined by calculating the relative dry weight to that of w/o channel hydrogel (%) post 2-week subcutaneous implantation in mice. Sample degradation was accelerated in vitro by immersing in PBS with stirring at 37 °C for 2 weeks. Samples were freeze-dried for 24 h and horizontally sectioned. The pore sizes of test groups were observed by field emission scanning electron microscopy (FE-SEM; MERLIN, Zeiss, Oberkochen, Baden-Württemberg, Germany), followed by quantitative analysis of the pore size using ImageJ/Fiji software.

### Laser Doppler imaging

Blood perfusion into the implant areas was determined at days 0, 7, and 14 post surgery by LDPI (Moor Instruments, Devon, UK) with quantitative analysis of LDPI values. The mouse body temperature was maintained by keeping it on a heating pad during LDPI scanning to minimize perfusion variations. The highest and lowest values of perfusion (LDPI) were color-coded to red and dark blue, respectively. The relative perfusion ratio was calculated by dividing the mean perfusion value of the ischemic hindlimb (left) by that of the normal hindlimb (right) from the corresponding LDPI image. Degrees of limb loss were determined following the reported amputation criteria in previous studies^55,56^. Tissue damage in the ischemic limb (limb salvage score) was graded as full recovery (grade 6), minor necrosis or nail loss (grade 5), partial toe amputation (grade 4), total toe amputation (grade 3), partial/total foot amputation (grade 2), or partial/total limb amputation (grade 1). Following these criteria, the damage degree of ischemic hindlimb tissue was quantitatively determined and presented as a limb salvage % among the ranges of limb rescue (recovery), toe loss, foot loss, and limb loss (amputation). Although the biological *N* (animal number) was 5, the degree of hindlimb ischemia salvage was scored by three blinded evaluators, and thus, the technical *N* was 11–14.

### MicroCT angiography

Blood perfusion from host blood vessels to each implanted hydrogel was determined by microCT angiography. After anesthetizing with isoflurane, the mice were subject to a tail vein injection of Pamiray 370 (100 µL of iopamidol-370, Dongkook CO., LTD, Republic of Korea) as a contrast agent and then to microCT scanning (NFR Polaris-G90, Nano Focus Ray, Korea) following a standard protocol (tube voltage: 65 Kvp; tube current: 115 µA; 720 views per 360° rotation; resolution: 100 μm). The acquired microCT images were reconstructed using the volumetric cone-beam reconstruction (FDK) algorithm, and analyzed using a 3D-rendering software (RadiAnt DICOM Viewer 4.2.1).

### In vivo EC infiltration and host vessel ingrowth

EC infiltration from the distal site of the ischemic hindlimb to the implanted hydrogels was determined by confocal imaging post tail vein injection of lectin (100 μL, FITC-conjugated, L-2895, Sigma–Aldrich) for 30 min. Flow perfusion connection from neighboring vessels to channel networks was imaged according to a previously reported method^57,58^. Briefly, after cutting the inferior vena cava of the mouse, PBS containing 0.1 mg mL^−1^ heparin sulfate was perfused through the left ventricle to remove whole blood through the cut drain point. PBS containing red fluorescent microbeads (45 nm in diameter, Invitrogen) was then perfused through the left ventricle for fluorescence visualization of perfusable vessels and channels, followed by sample harvest and fixation with 3.7% formaldehyde. Z-stacks of each whole-mount sample (gel with neighboring tissue) was subject to confocal imaging (LSM 780, Zeiss), followed by quantitative analysis of functional vasculature parameters (i.e. total branching length, branch number, junction number, and blood vessel fractional area per field of view) using ImageJ/Fiji software.

### Tissue and cell staining

The harvested samples were rinsed with PBS three times and then fixed with 10% weight volume^−1^ paraformaldehyde for 1 day, followed by embedding in paraffin to make sections. Sections of 4 µm thickness were obtained from six different locations of each block by microtome slicing, then subjected to staining with H&E and Masson’s trichrome for analysis of general histology and tissue fibrosis, respectively, by inverted microscopy (Leica DMi8, Leica Microsystems, Wetzlar, Germany).

The same sections were reused for immunofluorescence staining, and thus, hydrated by serial incubation in xylene and ethanol (100%, 95%, 80%, and 70% volume volume^−1^ in distilled water), followed by treatment with pepsin reagent (Sigma–Aldrich) for 30 min at 37 °C to retrieve antigens. The whole-mount sample was subject to blocking with a buffer solution of 5% weight volume^−1^ bovine serum albumin (Millipore) and 0.3% (weight volume^−1^) Triton X-100 (Sigma–Aldrich) for 1 h at room temperature. The sample blocks were then incubated with primary antibodies (1:100 dilution) for CD68 (ab125212, Abcam), CD31 (sc-1505, Santa Cruz Biotechnology), Involucrin (sc-21748, Santa Cruz), F4/80 (ab6640, Abcam), iNOS (ab955, Abcam), and CD206 (ab64693, Abcam) overnight at 4 °C, followed by incubation with Alexa Fluor 488 or Alexa Fluor 594-conjugated secondary antibodies (1:100 dilution) (Jackson Immuno Research Laboratories, West Grove, PA, USA). The sample blocks were counterstained with DAPI (H-1500, Vector Laboratories) and imaged by confocal microscopy (LSM 780, Zeiss) with quantitative analysis using ImageJ/Fiji software.

Cell apoptosis in samples was determined by TUNEL staining (Roche Molecular Biochemicals, Mannheim, Germany) according to the manufacturer’s protocol, followed by confocal imaging (LSM 780, Zeiss) with quantitative analysis using the ImageJ/Fiji software.

For whole-mount staining of the hydrogel, hydrogel samples were rinsed with PBS three times and then fixed with 10% paraformaldehyde for 1 day. The hydrogel samples were rinsed with PBS again and treated with primary antibodies (1:100 dilution) for CD31 (sc-1505, Santa Cruz Biotechnology, Dallas, Texas), CD34 (ab81289, Abcam, Cambridge, MA), CD133 (ab19898, Abcam) overnight at 4 °C, followed by treatment with Alexa Fluor 488 or Alexa Fluor 594-conjugated secondary antibodies (1:100 dilution; Jackson Immuno Research Laboratories, West Grove, PA). Cell nuclei were counterstained with DAPI (4′,6-diamidino-2-phenylindole; H-1500, Vector Laboratories) and imaged via confocal microscopy (LSM 780, Zeiss).

### Macrophage infiltration and polarization

In vivo monocyte infiltration to implantation sites was determined by labeling mouse monocytes (RAW264.7) with a fluorescent dye (Vivotrack 680, PerkinElmer, MA) according to the manufacturer’s instruction. These monocytes (2 × 10^6^ cells per mouse) were injected intravenously at day 3 post hindlimb ischemia surgery (*n* = 4), and their bio-distribution was determined using an IVIS (PerkinElmer) at 24 h post injection. The Vivotrack intensity at the gel implantation site of ischemic hindlimb was analyzed in comparison with that of the normal hindlimb. Samples were harvested post mouse euthanasia and processed for other studies.

In vitro macrophage polarization was determined by culturing RAW264.7 cells (5 × 10^5^ cells) in channel hydrogels with media perfusion at 20 μL min^−1^ by peristaltic pumping for 2 days. The samples were treated with LPS (100 ng mL^−1^) to induce inflammatory activation of RAW264.7 cells for 1 day and then subject to qRT-PCR analysis.

The effect of channel size on monocyte/macrophage polarization was confirmed by determining the migration and tubulogenesis of ECs under treatment of conditioned media. The conditioned media was obtained from a 2-day culture of RAW264.7 cells (5 × 10^5^ cells) within each hydrogel with a media perfusion at 20 μL min^−1^ via peristaltic pumping. As a control condition for M1 polarization, RAW264.7 cells were cultured under treatment of LPS (100 ng mL^−1^). For the tubulogenesis assay, human umbilical vein ECs (HUVECs, 2 × 10^5^ cells per well) were seeded onto matrigel (Corning, NY) in 24-well plates (ThermoFisher Scientific). For the migration assay, a PDMS disc (Dia. = 2 mm and thickness = 4 mm) was placed onto each of 24 wells, and the same number of HUVECs were seeded into the well such that HUVECs could migrate into the blank area where the PDMS disc was removed. Endothelial growth media (EGM-2, Lonza, Basel, Switzerland) was treated for 2 h to allow HUVEC attachment, followed by replacement of the media with the conditioned media from each test group. Tubulogenesis of HUVECs was imaged at 8 h post treatment of the conditioned media and analyzed using an angiogenesis analyzer plug-in at ImageJ/Fiji software. For the migration assay, PDMS disc was removed immediately after treatment of the conditioned media, and HUVEC migration into the blank area was then imaged at 8, 16, and 24 h post treatment of the conditioned media, so that the HUVECs’ covering area could be quantitatively determined.

### Quantitative RT-PCR analysis

Total RNA was prepared from implanted samples using TRIzol^®^ reagent and was then treated with DNase, followed by reverse transcription with RNA (1 μg) and random-hexamer primers to generate cDNA using a kit (TAKARA Bio Inc., Japan). Quantitative PCR was conducted with cDNA using SYBR^®^ Green and primers (forward and reverse) in a StepOne^TM^ Real-Time PCR system (Applied Biosystems, Foster City, CA). The relative gene expression level was determined by calculating the corresponding comparative Ct (2^−ΔΔCt^) value against that of glyceraldehyde 3-phosphate dehydrogenase (GAPDH) as a housekeeping gene. The primer sequences are listed in Supplementary Table 1.

### Statistical analysis

Each experiment was repeated three times. All data are presented as a mean ± SEM. All quantitative data were analyzed using a two-tailed Student’s *t*-test or one-way analysis of variance (ANOVA) with Tukey’s significant difference post-hoc test for multiple comparisons (SPSS 21.0K for Windows, SPSS, Chicago, IL, USA). Values of *p < 0.05, **p < 0.01, and ***p < 0.005 were considered statistically significant.

### Reporting summary

Further information on research design is available in the Nature Research Reporting Summary linked to this article.

## Supplementary information

Supplementary information

Reporting Summary

## Data Availability

The source data underlying Figs. 1b, d, e, g, and h, 2d–g, 3a, b, and d–g, 4a, 5b and d, 6e, and Supplementary Fig. 11a–c are provided as a Source Data file. All other relevant data are available upon request.

## Source data

Source Data
