# Supplementary material for: Microchannel network hydrogel induced ischemic blood perfusion connection
Source: Nat Commun. 2020 Jan 30;11:615. doi: 10.1038/s41467-020-14480-0 (PMC6992688; doi:10.1038/s41467-020-14480-0)
Supplement: Supplementary file 1 — Supplementary information [file 41467_2020_14480_MOESM1_ESM.pdf]

Supplementary Information for

Microchannel network hydrogel induced ischemic blood perfusion connection

Jung Bok Lee[1,5], Dae-Hyun Kim[1,5], Jeong-Kee Yoon[1,5], Dan Bi Park[1], Hye-Seon Kim[1], Young Min Shin[1], Wooyeol Baek[2], Mi-Lan Kang[1,3], Hyun Jung Kim[1,4], and Hak-Joon Sung[1,*]

[1]Department of Medical Engineering, Yonsei University College of Medicine, 50-1 Yonsei-ro, Seodaemun-gu, Seoul, 03722 Republic of Korea. [2]Department of Plastic & Reconstructive Surgery, Yonsei University College of Medicine, Seoul, Korea. [3] TMD LAB Co. Ltd, 50-1 Yonsei-ro, Seodaemun-gu, Seoul, 03722 Republic of Korea. [4] Department of Biomedical Engineering, University of Texas at Austin, Austin, TX, 78712, United States. [5]These authors contributed equally to this work

[*]To whom correspondence should be addressed. Email: hj72sung@yuhs.ac

This PDF file includes the following:

Supplementary Figures 1 to 11

Supplementary Tables 1

Supplementary Figures

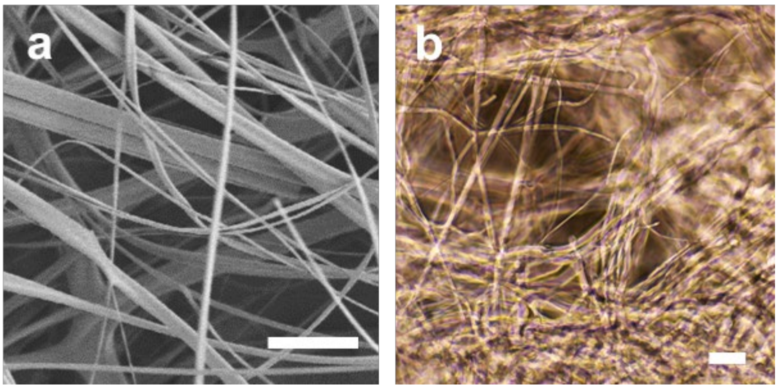

Supplementary Figure 1. **a** An SEM image of solvent spun PNIPAM fibres and **b** a bright field microscopic image of microchannel network hydrogel. Scale bars = 100 µm

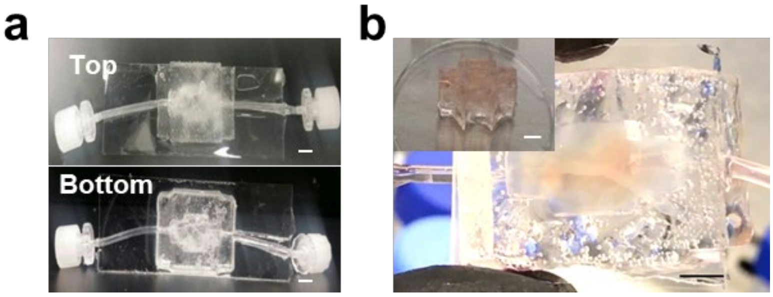

Supplementary Figure 2. **a** PNIPAM fibre-embedded gelatin hydrogel in a PDMS mould to generate microchannel networks. **b** Circulation of culture media through the microchannel network in a gelatin hydrogel. Scale bars = 5 mm

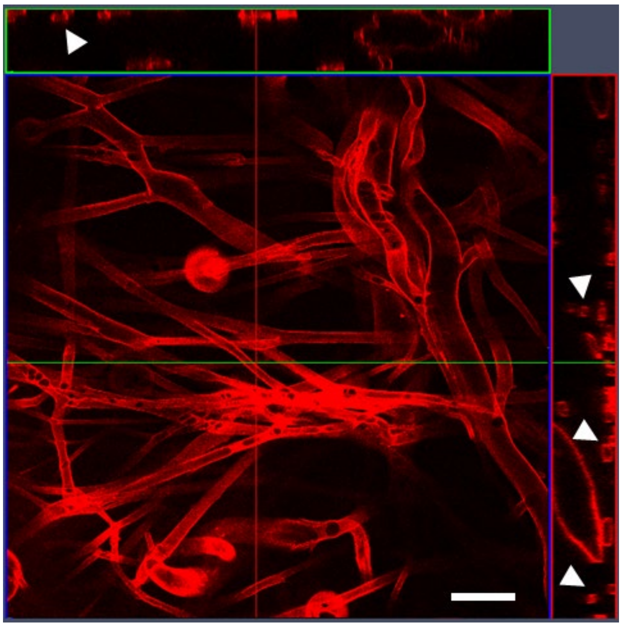

Supplementary Figure 3. High microchannel connectivity is demonstrated by the cross-sectional confocal image of gelatin hydrogel as 45 nm red FluoSpheres can be perfused only through the connected channels. White arrows indicate the channel lumens. Scale bar = 100 µm

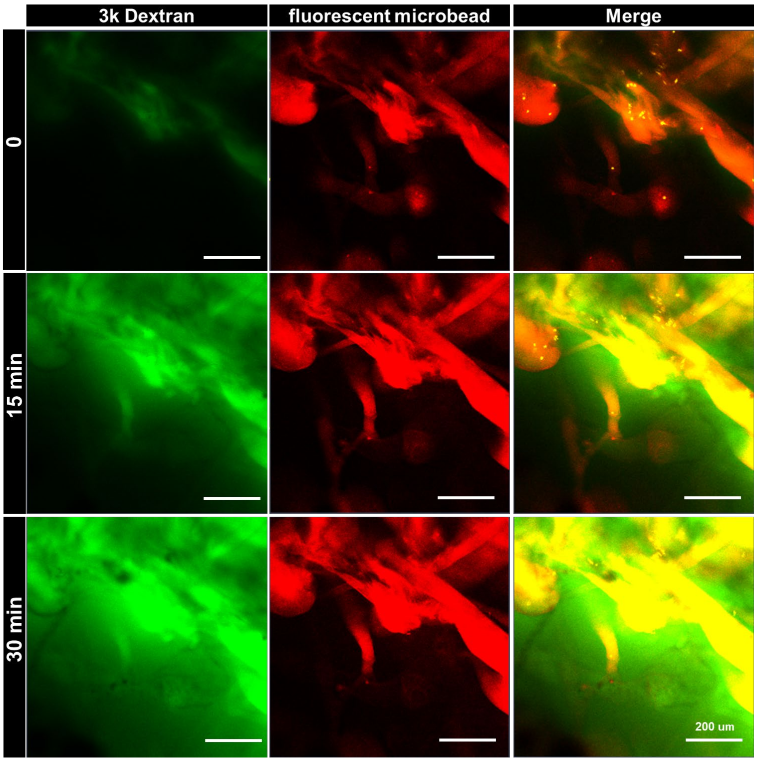

Supplementary Figure 4. Time-lapse confocal images. FITC-dextran (green) diffused efficiently from microchannels into the gelatin matrix in a hydrogel while microbeads (red) perfused through the microchannels well. Scale bar = 200 µm

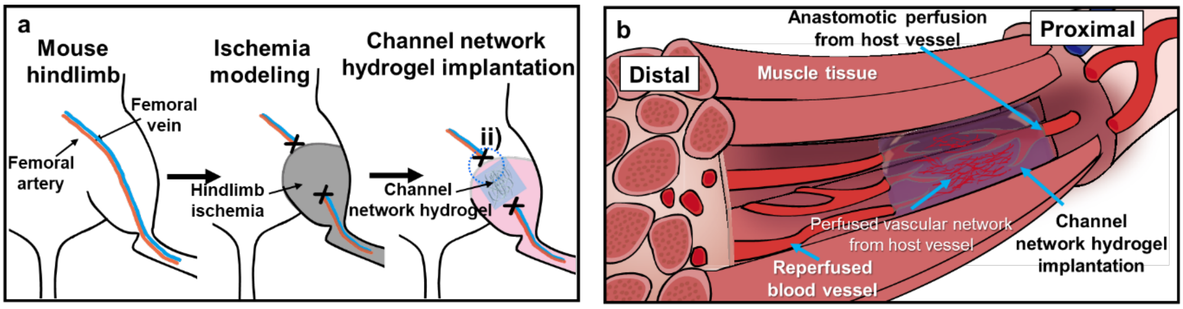

Supplementary Figure 5. Schematic demonstration of **a** hydrogel implantation into severely damaged skeletal muscle tissue post ligation and resection of the femoral artery/vein in a mouse model of hindlimb ischemia and **b** the discovered rescue mechanism by the ingrowth of host vessels and an established perfusion connection with channel networks in the implanted hydrogel.

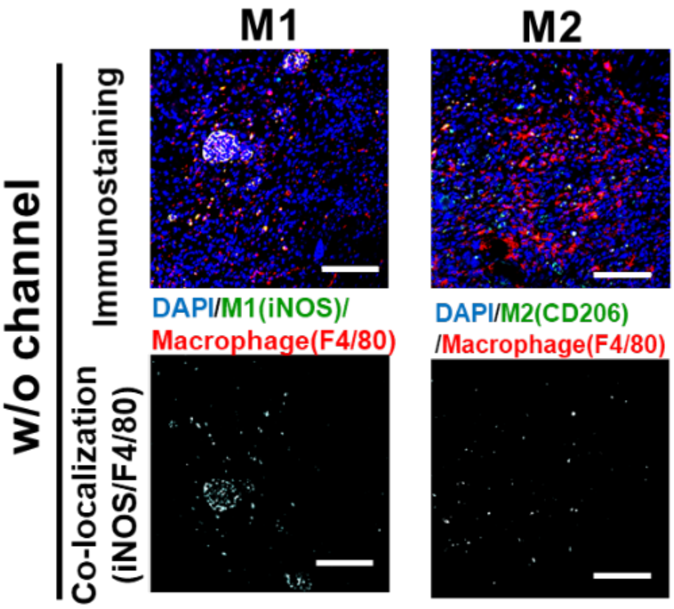

Supplementary Figure 6. Confocal images of mouse macrophages (red F4/80) with co-staining of polarization markers (M1: iNOS versus M2: CD206, both in green) in the w/o channel group (blue: nucleus staining with DAPI). Scale bar = 100 µm.

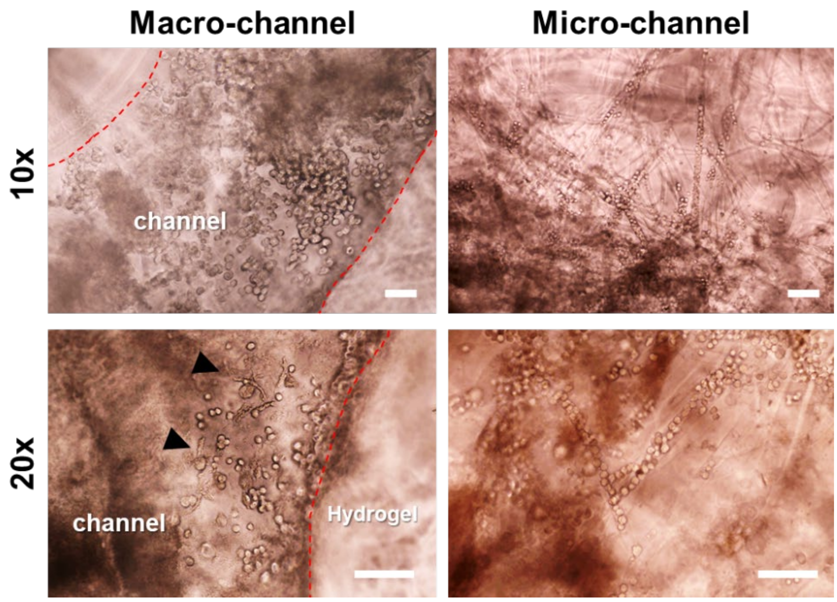

Supplementary Figure 7. Bright field microscopy images of mouse monocytes (RAW264.7) in the *in vitro* culture within macro- (left) and microchannel (right) network hydrogels. Adhered macrophages were not seen in microchannels, but were present in macro-channels (black arrows), indicating potential channel size-guided monocyte activation. Scale bar = 100 μm.

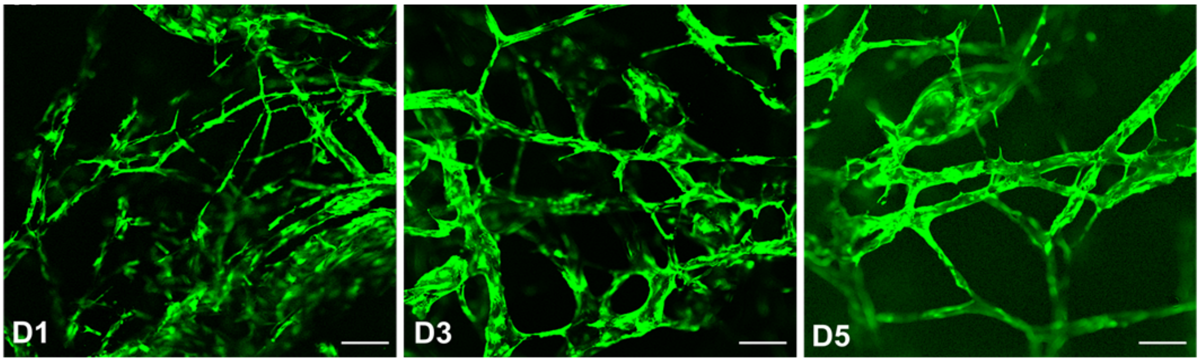

Supplementary Figure 8. Confocal images of *in vitro* GFP-HUVECs growth in a microchannel network hydrogel at days 1 (D1), 3 (D3), and 5 (D5) post culture with media perfusion. Scale bars = 100 μm

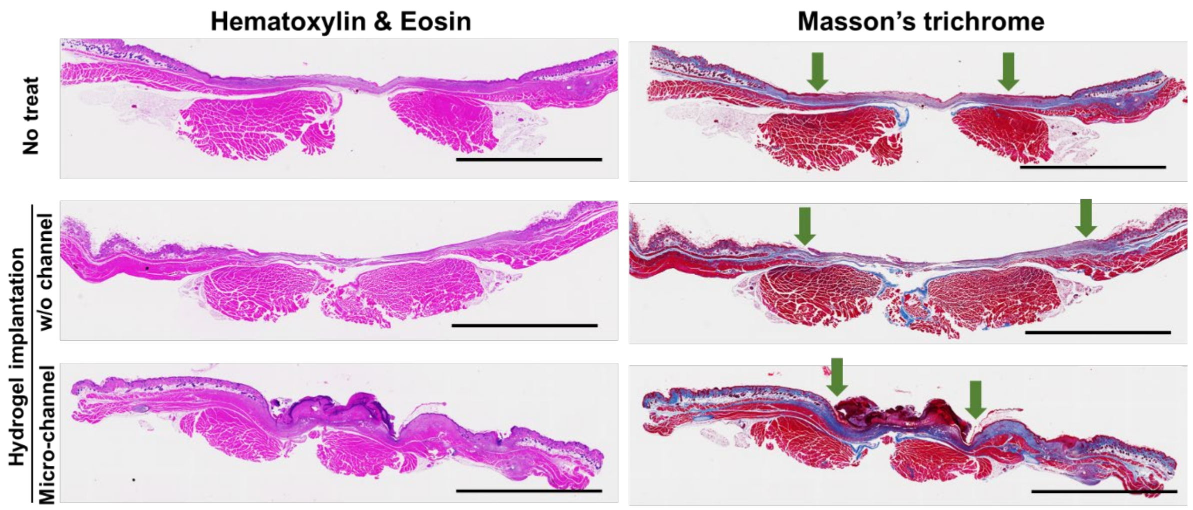

Supplementary Figure 9. General histology (H&E, left) and collagen formation (Masson's trichrome, right) of full-scale wound sites at day 14 post hydrogel implantation (Green arrow: a range of non-closed wound). Scale bar = 500 μm.

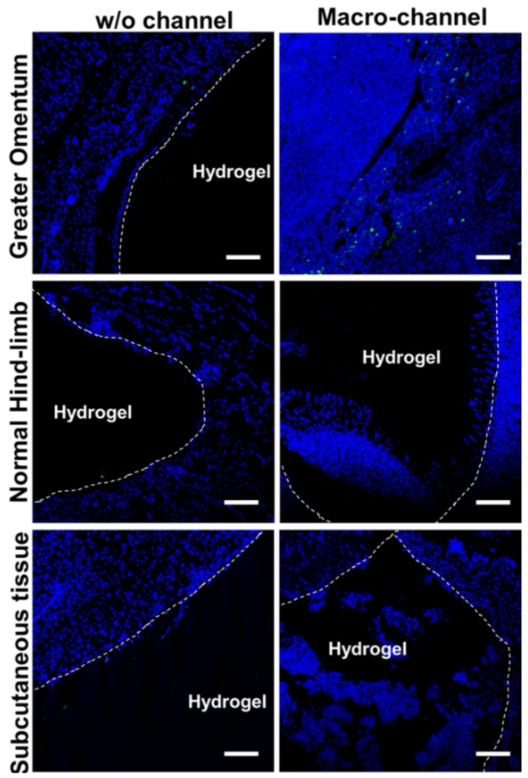

Supplementary Figure 10. Confocal microscopic images of CD 31^{+} cells (green) in the macro-channel network and w/o channel hydrogel groups (blue: nucleus staining with DAPI). Scale bar = 100 μm.

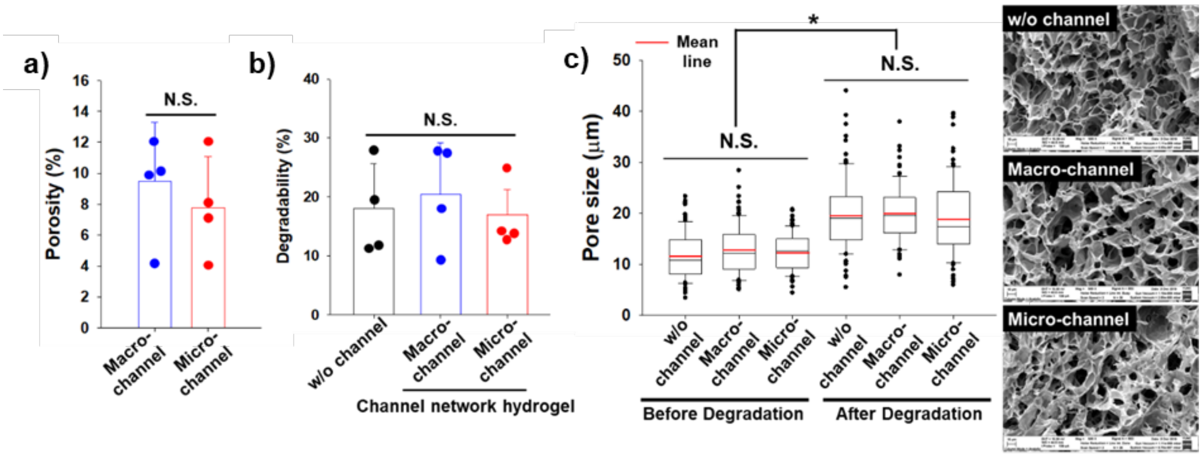

Supplementary Figure 11. Degradation effect on the porosity and pore size of test groups. **a** Porosity and **b** degradability of test groups (N = 4) at 2 weeks post subcutaneous implantation in mice. Dots represent each animal. Data are presented as mean ± SEM. (N.S.: not significant, one-way ANOVA with Tukey post-hoc pairwise comparisons) **c** Average pore size and cross-sectional SEM images of test groups before and after *in vitro* degradation. The sizes of 100 pores in SEM images (N = 4) of test groups were measured post 2-week accelerated degradation at 37°C. Box plots indicate the median and the first (25th Percentile) to third (75th Percentile) quartiles. The wihiskers denote 1.5 times the interquartile range. Boxplot outliers are shown in small black circles. *$p < 0.05$ between lined groups (N.S.: not significant) (one-way ANOVA with Tukey post-hoc pairwise comparisons). Source data are provided as a Source Data file.

Table S1. Primers used for qRT-PCR

Primer	Sequence (5′→3′)
**GAPDH**	Forward: ATGTGTCCGTCGTGGATCTGA<br>Reverse: TGCCTGTCTTCACCACCTTCT
**CD3**	Forward: GCGTCTGGTGCCTTCTTCAG<br>Reverse: AAAGTGTTCCACCGCATCCT
**CD68**	Forward: AAAGGCTTGGGGCATATCTGT<br>Reverse: GATGGGTACCGTCACAACCTC
**MyoG**	Forward: CCAAGTTGGTGTCAAAAGCC<br>Reverse: CAGGGCTGTTTTCTGGACATC
**αSMA**	Forward: CCTGGCCTAGCAACACTGATT<br>Reverse: AATTCTGTCACCCAGGGATGC
**KDR**	Forward: CATTGCCTGGTCAAACAGCTC<br>Reverse: TGGTGATCCTCTTGTAGCTCTCC
**vWF**	Forward: CAACTTGGAGCTATTGCAGGCAG<br>Reverse: CTTTCAGAGCCAGGGAGAGAAA
**CD31**	Forward: TCACCAAGAGAACGGAAGGC<br>Reverse: CTCTTCTCGGGACATGGACG
**CD34**	Forward: GTTGGGAAGAAAAGAGGCTGC<br>Reverse: GGGTTGTGAGGTACTGTGAGG
**CD133**	Forward: TGGAAGGAGCCCAGCTTAGA<br>Reverse: AGTACCATCCCTCTCCGGTC
**ARG1**	Forward: AGGGACTGACTACCTTAAACCAC<br>Reverse: TTCTGTCTGCTTTGCTGTGATG
**CD206**	Forward: TAGCACTGGGTTGCATTGGT<br>Reverse: TCCGCCAGGATAGTAAATGAGCA
**CD163**	Forward: TGCTGTCACTAACGCTCCTG<br>Reverse: CAGTTGTTTTCACCACCCGC
**IL10**	Forward: GGTTGCCAAGCCTTGTCTGA<br>Reverse: AGGGAGTTCACATGCGCCT
**IL1β**	Forward: GCCACCTTTTGACAGTGATGAG<br>Reverse: ATCAGGACAGCCCAGGTCAA
**IL6**	Forward: CAGGATACCACTCCCAACAGACC<br>Reverse: AAGTGCATCATCGTTGTTCATACA
