# Supplementary material for: Microchannel network hydrogel induced ischemic blood perfusion connection
Source: Nat Commun. 2020 Jan 30;11:615. doi: 10.1038/s41467-020-14480-0 (PMC6992688; doi:10.1038/s41467-020-14480-0)
Supplement: Supplementary file 2 — Reporting Summary [file 41467_2020_14480_MOESM2_ESM.pdf]

nature research

Corresponding author(s): Hak-Joon Sung

Last updated by author(s): Jan 2, 2020

Reporting Summary

Nature Research wishes to improve the reproducibility of the work that we publish. This form provides structure for consistency and transparency in reporting. For further information on Nature Research policies, see Authors & Referees and the Editorial Policy Checklist.

Statistics

For all statistical analyses, confirm that the following items are present in the figure legend, table legend, main text, or Methods section.

n/a	Confirmed	
☒	☐	The exact sample size (n) for each experimental group/condition, given as a discrete number and unit of measurement
☐	☒	A statement on whether measurements were taken from distinct samples or whether the same sample was measured repeatedly
☐	☒	The statistical test(s) used AND whether they are one- or two-sided<br>*Only common tests should be described solely by name; describe more complex techniques in the Methods section.*
☒	☐	A description of all covariates tested
☒	☐	A description of any assumptions or corrections, such as tests of normality and adjustment for multiple comparisons
☐	☒	A full description of the statistical parameters including central tendency (e.g. means) or other basic estimates (e.g. regression coefficient) AND variation (e.g. standard deviation) or associated estimates of uncertainty (e.g. confidence intervals)
☐	☒	For null hypothesis testing, the test statistic (e.g. F, t, r) with confidence intervals, effect sizes, degrees of freedom and P value noted<br>*Give P values as exact values whenever suitable.*
☒	☐	For Bayesian analysis, information on the choice of priors and Markov chain Monte Carlo settings
☒	☐	For hierarchical and complex designs, identification of the appropriate level for tests and full reporting of outcomes
☒	☐	Estimates of effect sizes (e.g. Cohen's d, Pearson's r), indicating how they were calculated

Our web collection on statistics for biologists contains articles on many of the points above.

Software and code

Policy information about availability of computer code

Data collection	Immunofluorescent staining images were quantified ZEN 2.3 SP1 (black, ver 14.0.0.0) and ImageJ/Fiji (1.52n)<br>IVIS images were quantified using Living Image software.<br>3D-rendering software (RadiAnt DICOM Viewer 4.2.1) was used for rendering of microCT images.
Data analysis	IMARIS (ver. 7.2.3), Sigmaplot (ver. 12.0), ZEN 2.3 SP1 (black, ver 14.0.0.0), ImageJ/Fiji (1.52n),RadiAnt DICOM Viewer (ver 4.2.1)

For manuscripts utilizing custom algorithms or software that are central to the research but not yet described in published literature, software must be made available to editors/reviewers. We strongly encourage code deposition in a community repository (e.g. GitHub). See the Nature Research guidelines for submitting code & software for further information.

Data

Policy information about availability of data

All manuscripts must include a data availability statement. This statement should provide the following information, where applicable:

- Accession codes, unique identifiers, or web links for publicly available datasets
- A list of figures that have associated raw data
- A description of any restrictions on data availability

The authors declare that all data supporting the findings of this study are available within the paper and its supplementary information files. Source data underlying Figures 1b, 1d-g, 2d-g, 3a, b, d-g, 4a, 5b, d, 6e and Supplementary Figures 11a-c are upon request from the corresponding author.

Field-specific reporting

Please select the one below that is the best fit for your research. If you are not sure, read the appropriate sections before making your selection.

☒ Life sciences ☐ Behavioural & social sciences ☐ Ecological, evolutionary & environmental sciences

For a reference copy of the document with all sections, see nature.com/documents/nr-reporting-summary-flat.pdf

Life sciences study design

All studies must disclose on these points even when the disclosure is negative.

Sample size	In most experiments, at least three samples were included in a single experiment, each with one to three technical replicates. Sample sizes were determined on the basis of our previous experience and of similar studies of other groups. For mouse in vivo studies, at least n=6 per group for hindlimb ischemia and n=4 per group for other experiments were selected on the basis of our prior experience of variability for these model system. For image quantification, at least three biological samples were used with one to three technical replicates. For porcine hindlimb ischemia model, total animal number was two and n=4 per each group were implanted.
Data exclusions	In the degree of hindlimb ischemia salvage test, ambiguous animals to score the salvage level were excluded.<br>For image analysis in tubulogenesis assay, images that include edge of well-plate were not included in the analysis.
Replication	Multiple samples at various times were analyzed and experiments were performed 3 times.
Randomization	All the allocation was random to minimize the biased effect to a specific sample.
Blinding	The degree of the damage in ischemic hindlimb tissue was blind evaluated by three evaluators. In preparation of samples for qPCR, researchers were not blinded, but investigators were blinded during data analysis.

Reporting for specific materials, systems and methods

We require information from authors about some types of materials, experimental systems and methods used in many studies. Here, indicate whether each material, system or method listed is relevant to your study. If you are not sure if a list item applies to your research, read the appropriate section before selecting a response.

Materials & experimental systems

n/a	Involved in the study	
☐	☒	Antibodies
☐	☒	Eukaryotic cell lines
☒	☐	Palaeontology
☐	☒	Animals and other organisms
☒	☐	Human research participants
☒	☐	Clinical data

Methods

n/a	Involved in the study	
☒	☐	ChIP-seq
☒	☐	Flow cytometry
☒	☐	MRI-based neuroimaging

Antibodies

Antibodies used	rabbit anti-CD68 antibody (ab125212, Abcam)<br>mouse anti-iNOS antibody (ab955, Abcam)<br>rabbit anti-CD206 antibody (ab64693, Abcam)<br>rat anti-F4/80 antibody (ab6640, Abcam red)<br>lectin (L-2895, Sigma Aldrich)<br>goat anti-CD31 antibody (sc-1505, Santa Cruz Biotechnology)<br>mouse anti-Involucrin (sc-21748, Santa Cruz Biotechnology)<br>Alexa fluor 488-conjugated anti-goat IgG (A11055, Life technologies)<br>Alexa fluor 488-conjugated anti-rabbit IgG (111-545-003, Jackson ImmunoResearch)<br>FITC-conjugated anti-mouse IgG (115-095-003, Jackson ImmunoResearch)<br>Alexa fluor 594-conjugated anti-rat IgG (712-585-150, Jackson ImmunoResearch)
Validation	There is no novel antibodies in this study. All antibodies used were well described in literature or in the manufacturer's protocols. The protocols for antibody dilution and incubation time in the immunofluorescence assay were optimized.

Eukaryotic cell lines

Policy information about cell lines

Cell line source(s)	RAW264.7 cells: ATCC<br>GFP expressing human umbilical vein endothelial cell (GFP-HUVEC): ANGIO-PROTEOMIE
Authentication	None of the cell lines used were authenticated
Mycoplasma contamination	The cell lines were not tested for mycoplasma contamination
Commonly misidentified lines<br>(See ICLAC register)	None

Animals and other organisms

Policy information about studies involving animals; ARRIVE guidelines recommended for reporting animal research

Laboratory animals	Mouse: Balb/c (Male, 5 weeks)<br>Porcine: Yorkshire pig (Female, 3 months, 25-30kg)
Wild animals	None
Field-collected samples	None
Ethics oversight	All animal studies were carried out by procedures approved by the Institutional Animal Care and Use Committee of Yonsei University College of Medicine (2016-0194 and 2017-0058 for mouse and porcine, respectively). . Four Mmice were grouped to housed in groups of four, in each wire-mesh cages [(W)200 × (D)260 × (H) 130 mm] and housed in a temperature (22 ± 2 °C) and humidity (50 ± 10%) regulated environment with 12-hour light-dark cycle.

Note that full information on the approval of the study protocol must also be provided in the manuscript.
